# Evaluation of the Sustainability of Marine Resources in the Reef Areas of Furong Island Marine Ranching Based on the Ecopath Model

**DOI:** 10.3390/biology15181620

**Published:** 2026-09-15

**Authors:** Xin Tong, Ang Li, Ling Zhu, Jiamin Zhang, Hongbin Han, Xuan Ding, Yanan Jiang, Yuze Mao

**Affiliations:** 1 State Key Laboratory of Mariculture Biobreeding and Sustainable Goods, Yellow Sea Fisheries Research Institute, Chinese Academy of Fishery Sciences, Qingdao 266071, China; tongxin_qd@163.com (X.T.); lia@ysfri.ac.cn (A.L.); maoyz@ysfri.ac.cn (Y.M.); 2 Laboratory for Marine Ecology and Environmental Science, Qingdao Marine Science and Technology Center, Qingdao 266237, China; 3 Laizhou Marine Development and Fishery Service Center, Laizhou 261400, China; 17663887198@163.com (J.Z.); 15264197821@163.com (H.H.); 15688568168@163.com (X.D.); jiang1073085181@163.com (Y.J.)

**Keywords:** marine ranching, sustainable conservation, ecopath model, reef deployment

## Abstract

Marine ranching is a crucial component of the “blue granary” strategy. Evaluating the long-term conservation effectiveness of artificial reef areas in marine ranches helps to assess the current conservation status, identify issues during the conservation process, and develop targeted solutions. Based on survey data from the national marine ranching in the western waters of Furong Island in 2019 and 2022, we used Ecopath with Ecosim version 6.7 to construct Ecopath models for the two periods. By comparing factors such as trophic structure, system attributes, and energy flows, we evaluated the potential ecological effects associated with sustained conservation and management activities. The results indicated that the trophic level structures of the ecosystem food web between 2019 and 2022 were similar. The trophic levels of most functional groups in 2022 were slightly higher than those in 2019, and the biomass increased significantly. The biodiversity slightly increased, with the Shannon index rising from 1.544 to 1.570, and the trophic levels of most functional groups increased. The overall ecosystem scale expanded by 29.37%, while total primary production/total respiration (TPP/TR), total primary production/total biomass (TPP/TB), and the ascendancy (A) value decreased, and Finn’s cycling index (FCI), mean path length (MPL), and transfer efficiency (TE) increased. Collectively, these changes suggest a tendency toward greater ecosystem maturity, although not all ecosystem indicators exhibited consistent responses. In the energy transfer process, the proportion of energy flowing into each trophic level improved. The detrital food chain dominated the system’s energy supply, though its proportion of total flow decreased from 61% to 57% over the years, while the energy contribution from primary producers increased, and the total transfer efficiency of both pathways improved. Analysis of mixed trophic impacts and keystone species revealed changes in keystone species. Overall, the observed changes from 2019 to 2022 were consistent with potential ecological improvements under sustained conservation and management. These findings will contribute to the further refinement of fisheries resource conservation methods and support decision-making for marine ecosystem restoration.

## Simple Summary

Marine ranching, particularly through artificial reef deployment and associated conservation practices, is widely used to restore marine habitats and conserve fishery resources. However, the long-term effectiveness of these measures in sustaining and restoring marine fishery resources requires systematic evaluation at the ecosystem level. In this study, we compared the food-web structure and energy flow of the Furong Island marine ranching ecosystem in 2019 and 2022 using an Ecopath model. Most functional groups showed higher biomass in 2022, while the overall ecosystem scale and energy transfer efficiency also increased. Several ecosystem indicators generally suggested a tendency toward greater ecosystem maturity and more efficient internal energy use, although not all indicators changed consistently. Detritus remained the main source of energy supporting the food web, while the contribution of primary producers increased. Changes in species interactions were also observed between the two years. Overall, these findings are consistent with potential ecological improvements under sustained conservation and management.

## 1. Introduction

Fisheries resources serve as a critical foundation for global food security and the core driver of the blue economy, providing at least 20% of per capita protein intake for 3.2 billion people worldwide, thus representing a strategic asset for the sustainable development of coastal nations [1,2]. However, global fishery resources have faced severe declines due to the combined impacts of climate change, overfishing, and environmental pollution, leading to widespread ecosystem degradation [3]. In response to these challenges, marine ranching has emerged as a vital component of the national “blue granary” strategy, playing an irreplaceable role in habitat restoration and resource conservation [4,5].

Artificial reefs serve as foundational structures and key engineering tools within marine ranching. By providing shelter, foraging grounds, and spawning sites, they mitigate the impacts of currents and predators, thereby supporting ecological restoration and fishery conservation [6,7,8]. Studies have shown that artificial reefs alter local hydrodynamics, enhance nutrient enrichment [9,10], and promote biomass accumulation and biodiversity [11,12]. However, most existing research has focused on short-term effects before and after reef deployment, with limited attention to the long-term sustainability of resource conservation. Given that fisheries restoration is a protracted process, establishing a robust framework for evaluating sustained conservation effectiveness is essential for refining management practices and promoting the healthy development of marine resources.

Ecosystem structure is shaped by complex interactions such as predation, competition, and symbiosis, which govern energy flow and material cycling. Environmental disturbances can alter these ecological networks, leading to structural and functional shifts [13]. Model simulations offer a simplified yet powerful approach to capture ecosystem dynamics. Among these, the Ecopath with Ecosim (EwE) model has become a widely used tool for analyzing marine food webs and energy flows [14]. Initially developed by Polovina and refined by Christensen and Pauly [15,16], the Ecopath model provides a static representation of trophic interactions and system stability, making it suitable for assessing ecological carrying capacity and ecosystem responses to changes in species biomass [3,17].

Laizhou Bay, one of three major bays of the Bohai Sea, is an important spawning and nursery ground for commercially valuable species in northern China [18,19]. However, intensive human activities have led to a decline in fishery resources, reduced biodiversity, and a shift toward smaller, lower-trophic-level organisms [19]. To address these issues, marine ranching has been established in Laizhou Bay, contributing to local ecological environment improvement. Recent assessments indicate that ecosystem health has transitioned from “unhealthy” to “sub-healthy”, with increases in biodiversity indices for phytoplankton, zooplankton, and benthos [20]. Systematic evaluation and targeted restoration remain imperative for further ecological recovery.

This study aims to evaluate the potential positive effects of sustained conservation on the resources of the artificial reef area in the national marine ranching west of Furong Island, Laizhou Bay, from 2019 to 2022. Using the Ecopath model, we analyzed energy flow, trophic structure, and system maturity under continuous conservation. We also identified keystone species through mixed trophic impact and keystone index analyses. The study evaluates the long-term effectiveness of resource conservation and provides a scientific basis for the management and sustainable use of marine ranching ecosystems.

## 2. Materials and Methods

### 2.1. Study Area

**Figure 1:**
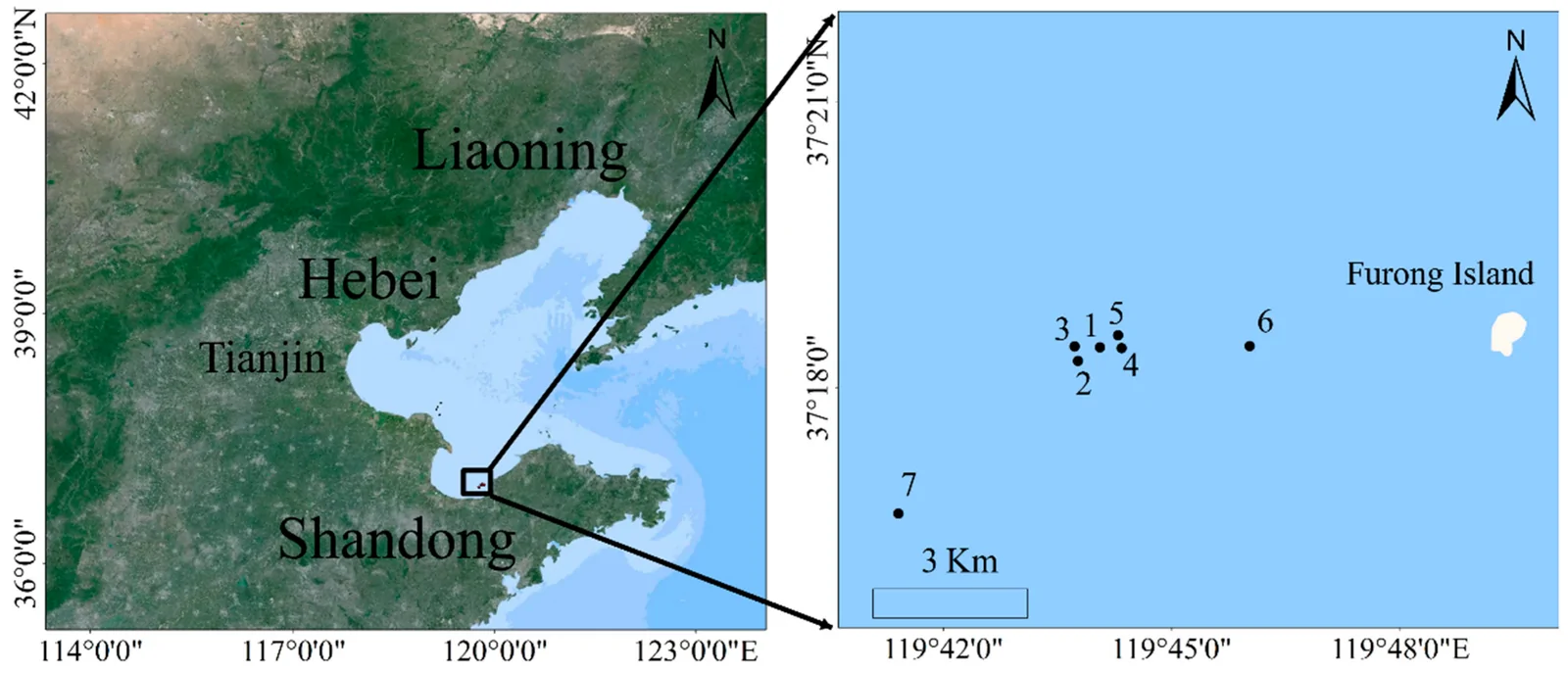
Sampling stations in Furong Island. Numbers 1–7 indicate the locations of Stations 1–7.

The study area is located in the national marine ranching demonstration area in the western waters of Furong Island in Laizhou Bay, with a total area of 107.95 km^2^ and a core area of 33.3 km^2^ (37°14′33.72″–37°21′59.65″ N, 119°36′01.80″–119°45′03.92″ E), as shown in Figure 1. The ranching was built by Shandong Blue Ocean Technology Co., Ltd. in 2015. The construction projects include the deployment of artificial fish reefs, transplantation and cultivation of mixed seaweed beds in seaweed fields, bottom sowing, and stock enhancement. The artificial fish reefs comprise 2.20% precast reinforced concrete and 97.80% stone, totaling 1.408 million cubic units; the seaweed fields focus on *Gracilaria lemaneiformis* transplantation, with an area of approximately 0.16 km^2^; shrimps, crabs, fingerlings, and cuttlefish are released to bolster fishery resources. The primary ecological function of the ranching is resource and ecological protection.

During October–November 2019 and October 2022, we set up 7 stations in the ranching and the adjacent sea area respectively. Data were collected on both physicochemical parameters of the water column and marine biological elements. The sampling stations (37°16′42″–37°18′39″ N, 119°41′21.6″–119°45′54″ E) are shown in Figure 1.

### 2.2. Ecopath Model

We utilized Ecopath with Ecosim version 6.7 (http://www.ecopath.org/, accessed on 16 October 2025) to construct ecosystem models for the Furong Island marine ranching. These models quantitatively depict the energy flow process within the ecosystem. Based on the law of conservation of energy, the formula used is as follows:

(1) $$B_{i}\times \left(P/B\right)\times EE_{i}-\sum_{j=1}^{n}B_{j}\times {\left(\frac{Q}{B}\right)}_{i}\times DC_{ji}-Y_{i}-E_{i}-BA_{i}=0$$

where *B_i_* and *B_j_* are the biomass of functional group *i* and its predator *j*, respectively, (*P*/*B*)*_i_* is the ratio of production to biomass for functional group *i*, (*Q*/*B*)*_i_* is the ratio of consumption to biomass for functional group *i*, *DC_ji_* is the proportion of prey species *j* in the diet composition of predator species *i*, $Y_{i}$ is the catch of functional group *i*, *BA_i_* is the accumulated biomass of functional group *i,* and *E_i_* is the output of functional group *i*.

The parameters required for constructing the Ecopath model include *B_i_*, (*P*/*B*)*_i_*, (*Q*/*B*)*_i_*, ecotrophic efficiency (*EE_i_*), diet composition (*DC_ij_*), and fishing catch. Given any three of the first four parameters, the fourth one can be calculated. Since the main function of the study area is conservation, fishing catch is not considered.

We use both trophic level and mean trophic level for illustration. The trophic level is automatically generated by the model, and the mean trophic level was calculated based on the method described by Jennings and Blanchard [21]. The calculation formula is as follows:

$${TL}_{m}=\frac{\sum {TL}_{ij}B_{ij}}{\sum B_{ij}}$$

where $\;{TL}_{ij}\;$ is the trophic level of species *i* in year *j*, and $B_{ij}$ is the biomass of species *i* in year *j*.

### 2.3. Functional Group Classification and Input Parameters

In accordance with the theory of nutritional dynamics, and similar feeding habits, ecological characteristics, and significance among biological species, the reef deployment area of the Furong Island marine ranching ecosystem in 2019 and 2022 was categorized into 16 and 17 functional groups respectively. This classification includes key fishery populations, macro-benthos, zooplankton, phytoplankton, and detritus (Table 1). These functional groups are linked through trophic relationships, covering the entire energy flow from low to high trophic levels.

The input parameter values of each functional group were determined as the weighted average of the values within the group, with weights based on the relative biomass of each group. Nekton including fish, shrimps, crabs, and cephalopods were collected using ground cages with a length of 7.8 m, a total of 20 sections, each section measuring 24 cm × 30 cm × 18 cm, and a mesh size of 1 cm × 1 cm. Benthic biological samples were obtained using a 0.05 m^2^ model sediment sampler. Phytoplankton biomass was analyzed via the spectrophotometric method, and the chlorophyll a content was calculated using the Jeffrey-Humphrey equation. Biomass was then determined using formulas incorporating factors such as carbon units and water depth [22,23,24,25,26,27]. Among them, the ratio of phytoplankton (carbon units) to chlorophyll a was selected as 56 µgµg^−1^, which was an average value [28]. The detritus biomass was calculated by referring to existing studies [27,29,30,31]. The calculation Formulas (2)–(4) are as follows:

*E* = 3.05*D_t_* (2)

lg*P* = 3 + 0.5lg*C*_a_ (3)

lg*D_b_* = −2.41 + 0.954lg*PP* + 0.863lg*E* (4)

where *E* is the euphotic depth (m), *D_t_* is the transparency (m), *P* is the daily primary productivity (mg C/m^2^·d), *C*_a_ is the surface chlorophyll a content (mg/m^3^), *PP* is the annual primary productivity (mg C/m^2^·a), *D_b_* is detritus biomass (g C/m^2^).

The biomass of *Rapana venosa*, *Apostichopus japonicus*, oysters, and zooplankton was referenced from relevant literature [12,32].

The P/B and Q/B values for individual functional groups were obtained from analogous groups in studies of ecologically similar ecosystems. For aggregated functional groups, these parameters were further calculated through weighted averaging [12,33,34].

The diets of individual functional group were obtained from literature on stomach contents studies [32,35,36,37,38,39,40], and aggregate functional groups diets were derived by weighting relevant studies [12,32,36,37,38,40,41,42].

In these models, the unit of biomass flow was t·(km^−2^·a), and biomass was t·km^−2^. Since ecotrophic efficiency (EE) was difficult to obtain directly, most EE values were calculated automatically by the Ecopath model. The unassimilated amount was set as 0.4 for oysters, 0.35 for other macro-benthic animals, 0.4 for zooplankton, and 0.2 for the remaining groups. The detritus input in the reef deployment area was 433.84 t·(km^−2^·a) in 2019 [12] and 320 t (km^−2^·a) in 2022 [32].

### 2.4. Model Validation and Calibration

The model may be unbalanced during its initial run. Prior to model balancing, it must be tested using the pre-balancing diagnostic method (PREBAL) [43]. Based on ecological principles and empirical rules, this method systematically examines the slope characteristics of biomass (B), production/biomass (P/B), and consumption/biomass (Q/B) as they vary across trophic levels [14]. After taking logarithms of B, P/B, and Q/B respectively, the slopes of biomass (log scale) for each trophic level should typically decrease by 5–10% relative to the 10% slope baseline. This benchmark was used to assess the deviation of each functional group’s data from the expected line and to review the accuracy and completeness of data for functional groups that showed significant deviations [43].

**Figure 2:**
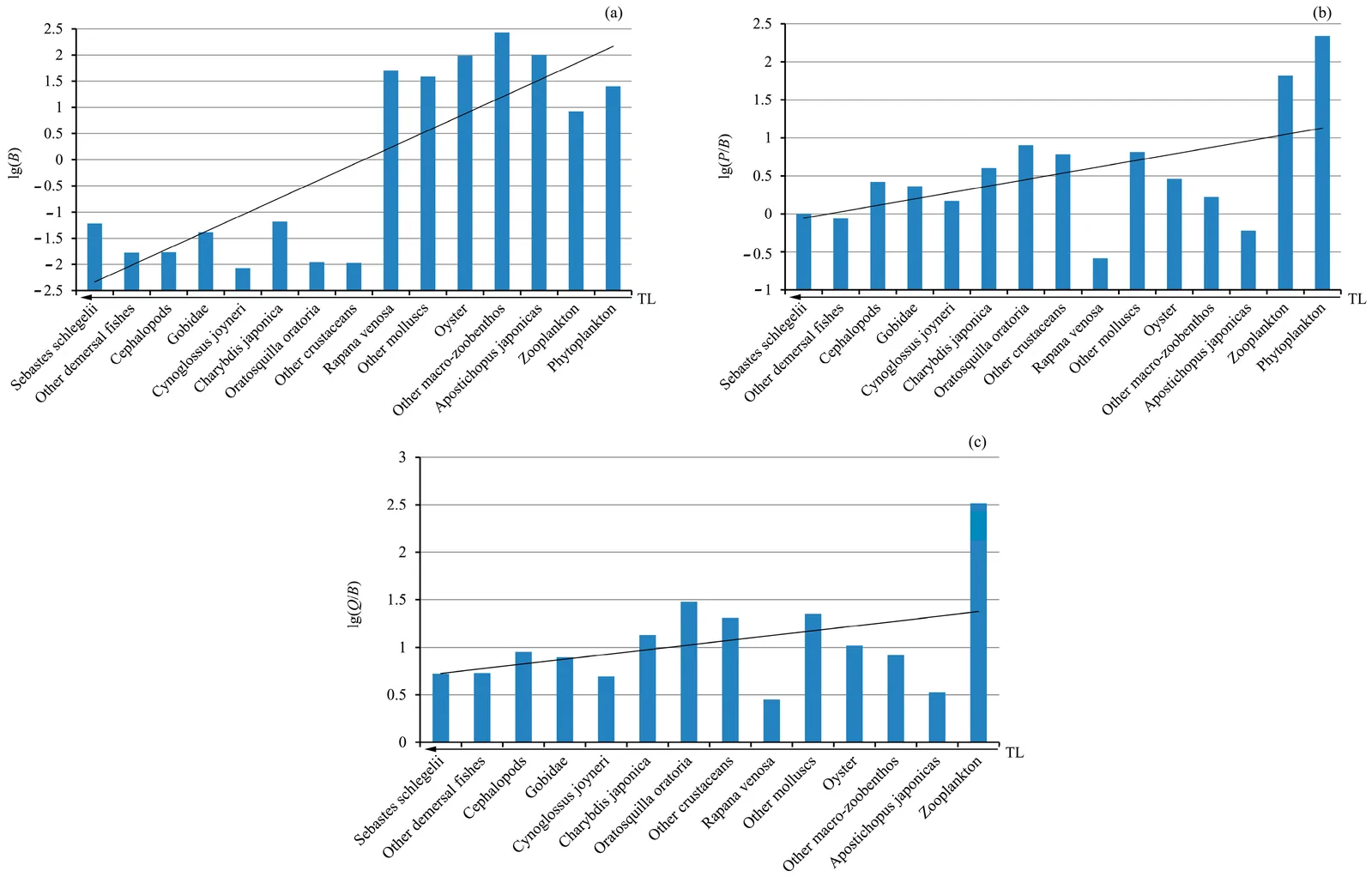
(**a**) Prebal analysis of the 2019 log biomass estimate (t·km^−2^). (**b**) Prebal analysis of the 2019 log P/B estimate. (**c**) Prebal analysis of the 2019 log Q/B estimate.

**Figure 3:**
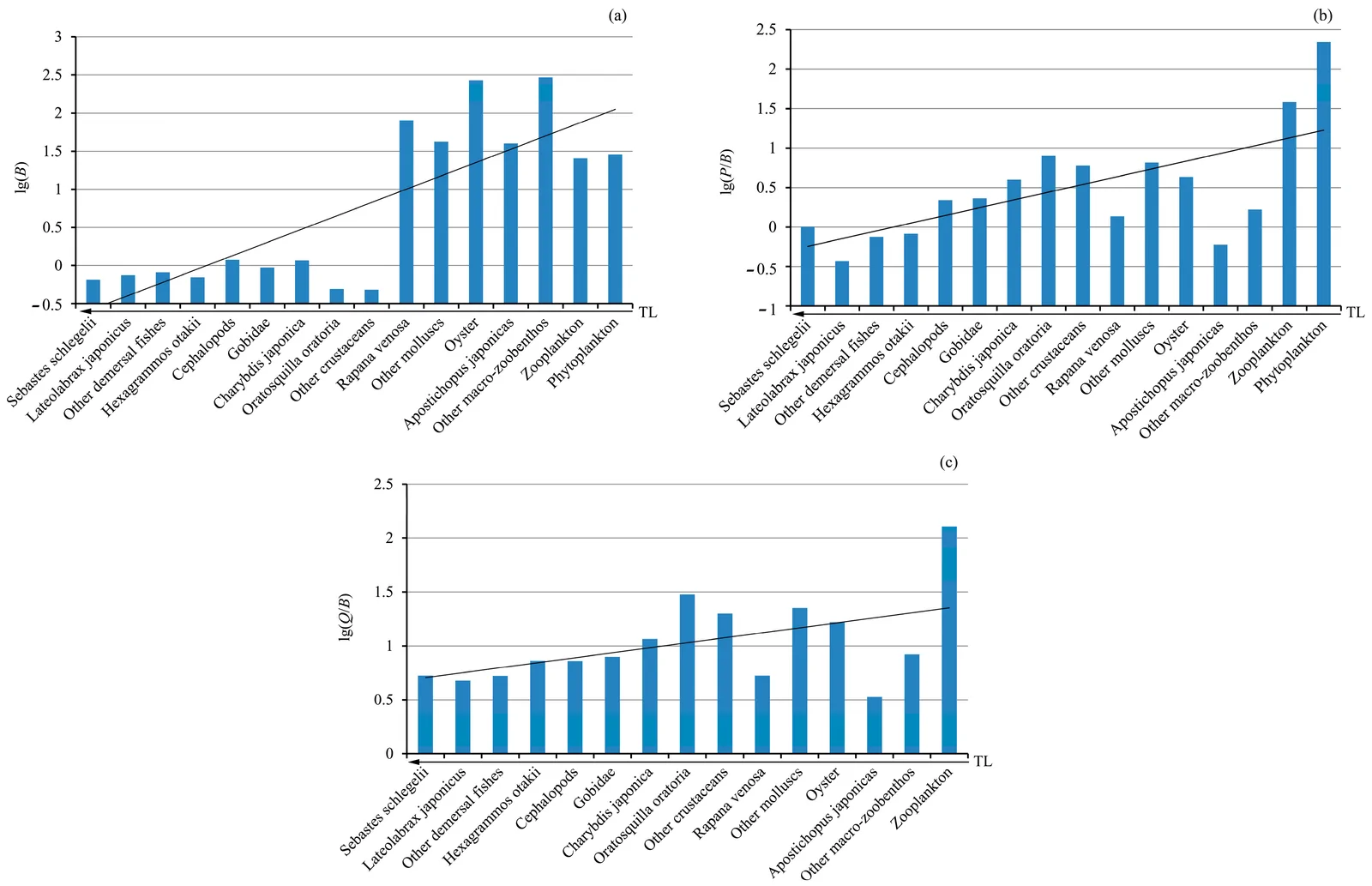
(**a**) Prebal analysis of the 2022 log biomass estimate (t·km^−2^). (**b**) Prebal analysis of the 2022 log P/B estimate. (**c**) Prebal analysis of the 2022 log Q/B estimate.

The pre-balancing results are shown in Figure 2 and Figure 3. During model construction, deviations from the diagnostic criteria were assessed, and parameters were reset when necessary. Following PREBAL diagnostics, the model underwent equilibrium validation according to thermodynamic and ecological principles proposed by Darwall et al. [44], including (1) Ecological efficiency (EE) of all components satisfies 0 < EE < 1.0; (2) P/Q ratios for most functional groups range between 0.1 and 0.3; (3) Production/respiration ratio is less than 1 but approaches 1 as the system matures.

To meet the above requirements and achieve model equilibrium, adjustments were made to the P/B, Q/B, and diet matrix. If the model remained unbalanced, biomass was also adjusted, particularly when data originated from areas outside the modeling region [45].

Because some input parameters were derived from previous studies of comparable ecosystems, the reliability of the data sources and the plausibility of the model inputs were evaluated using the Pedigree index. Morissette reported that Pedigree index values of previously developed Ecopath models generally ranged from 0.164 to 0.676. The quality of model input data can be further categorized into four levels: low (<0.20), moderate (0.20–0.399), high (0.40–0.599), and very high (≥0.60) [46]. Accordingly, the Pedigree index calculated for the present model was used as an indicator of the overall quality, reliability, and appropriateness of the input data.

### 2.5. Ecosystem Attributes

Ecopath includes multiple ecological indicators to describe the stability and maturity of the ecosystem being modeled under current conditions.

Total system throughput (TST) reflects the scale and metabolic capacity of the system [47]. It is the sum of total consumption (TC), total exports (TE), total respiration (TR), and total flow into detritus (TD) [48].

Total primary production/total respiration (TPP/TR), total primary production/total biomass (TPP/TB), and total biomass/total throughput (TB/TST) characterize system maturity. As systems mature, TPP/TR gradually approaches 1 [49], TPP/TB values decrease [49], and TB/TST values increase [48].

Net system production (NSP) also serves as an indicator of ecosystem maturity to a certain extent. A lower NSP value indicates a more mature ecosystem [49].

The Connectance Index (CI) and System Omnivory Index (SOI) reflect the complexity of food webs within a system. Values closer to 1 for both indexes indicate a more developed system with more complex feeding relationships [48].

Finn’s Cycling Index (FCI) and the Mean Path Length (MPL) represent the proportion of recycled flow to the total system throughput and the average length of all potential pathways in the food web, respectively. Mature systems exhibit higher recycling rates and longer path lengths than immature systems [49].

Ascendancy (A) reveals the trade-off between the orderliness of energy flows and ecosystem stability. Higher values indicate greater conversion efficiency but also greater system vulnerability, while lower values signify stronger system resilience against disturbances. Its trend exhibits a negative correlation with system maturity [47,49].

Mixed trophic impact can be observed through changes in the biomass of functional groups, revealing the effects on other functional groups [50]. This reflects the predation relationships and interactions among functional groups within the model [48].

## 3. Results

### 3.1. Model Robustness and Trophic Level Characteristics

The Pedigree index values for the two models were 0.638 and 0.661, respectively, indicating very high quality and reliability of the model input data.

The input parameters of the Ecopath model and the basic parameters after debugging and balancing are shown in Table 2. The diet compositions are shown in Tables S1 and S2. The EE value ranges of 2019 and 2022 in the model are 0–0.959 and 0–0.989, respectively. The trophic structures of the ecosystem food webs in the 2019 and 2022 survey periods were similar. The trophic level ranges of 2019 and 2022 are 1–4.079 and 1–4.183 respectively, and their mean trophic levels were 2.050 and 2.159, respectively.

The functional group with the highest trophic level was *Sebastes schlegelii*. The trophic levels of most functional groups were slightly higher in 2022 than in 2019, and several functional groups showed substantially higher biomass. The ecological trophic efficiencies of the functional groups at the highest trophic level were relatively low, at 0.325 and 0.393, respectively.

In 2019 and 2022, trophic level I in Furong Island marine ranching comprised phytoplankton and detritus, while trophic level II included zooplankton, some zoobenthos, and some mollusca, serving as conduits for upward energy transfer in the food web. When primary productivity increases, the biomass of primary consumers feeding on primary producers expands due to the bottom-up effect. This may provide a larger food supply for higher-trophic-level predators and could be associated with the higher biomass estimated for some upper-trophic-level groups. As shown in Table 2 and Table 3, phytoplankton biomass and total primary production both increased by 13.78% in 2022. Among them, the biomass of phytoplankton increased from 25.206 t km^−2^ to 28.68 t km^−2^, and the total primary production increased from 5545.32 to 6309.600 t·km^−2^·a^−1^. Except for *Apostichopus japonicus*, whose biomass declined to approximately 40% of its 2019 level, the other primary consumers showed increasing biomass. Consequently, the biomass of the top predator *Sebastes schlegelii* increased from 0.0605 t·km^−2^ to 0.6537 t·km^−2^, representing a 10.8-fold increase. This pattern may indicate that bottom-up processes contributed to the recovery of high-trophic-level fish resources.

### 3.2. Changes in Ecosystem Attribute Parameters

Table 3 displays the characteristic parameters of the energy flow in the reef deployment area ecosystems in 2019 and 2022. In 2022, the TST changed from 20,368.160 t·km^−2^·a to 26,351.131 t·km^−2^·a, with an increase of approximately 29.37% in scale. The increase in TST was mainly driven by the rise in total consumption (TC), which increased by 4388.378 t·km^−2^·a^−1^ and accounted for approximately 73.35% of the net increase in TST. Total respiration (TR) and flows to detritus (TDET) also contributed to the increase, accounting for approximately 20.84% and 12.04%, respectively. In contrast, total export (TEX) decreased by 372.601 t·km^−2^·a^−1^, partially offsetting the increase in TST. Therefore, the expansion of ecosystem throughput was primarily associated with enhanced internal consumption rather than increased export.

Compared with 2019, the values of TPP/TR, TPP/TB, CI, and A decreased in 2022, while the values of FCI, MPL, SOI, and TE increased.

**Figure 4:**
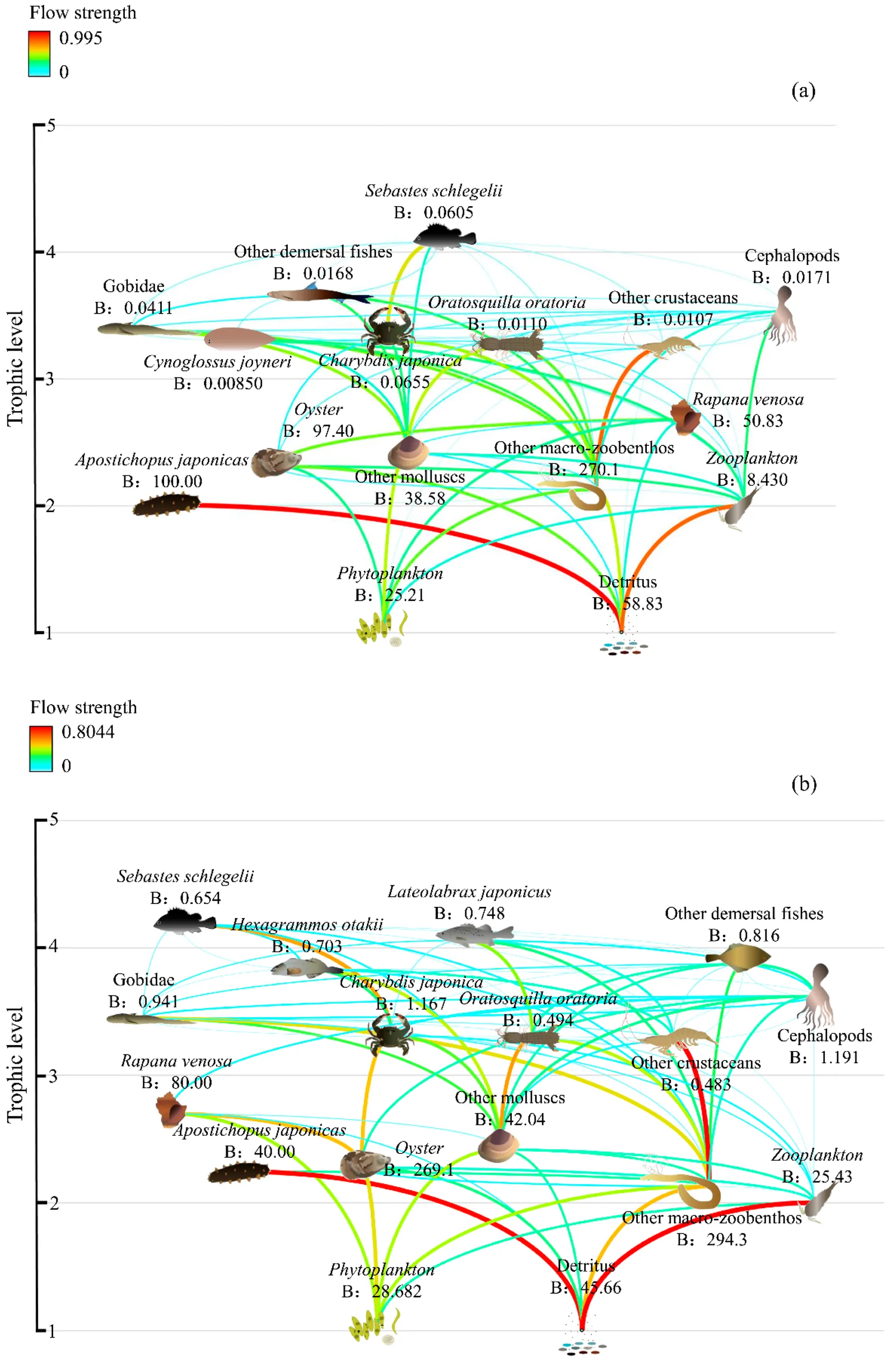
(**a**) The energy flow diagram of the reef deployment area in 2019. (**b**) The energy flow diagram of the reef deployment area in 2022. Note: The colors in the figure represent the energy flow intensity along the connecting lines between functional groups, with energy flow increasing from blue to red. The vertical axis denotes trophic levels, and the B values in the figure indicate the biomass of each functional group.

The energy flow and biomass among functional groups of the reef deployment area in 2019 and 2022 are shown in Figure 4. In each of the two years after reef deployment, two distinct food chains emerged: a phytoplankton-based grazing food chain and a detritus-based detrital food chain. The grazing food chain originates from phytoplankton, transferring energy upward through zooplankton, mollusca, and benthic organisms to reach the top consumers, *Sebastes schlegelii* and *Lateolabrax japonicus.* The primary consumer in the detrital food chain is *Apostichopus japonicus*, followed by zooplankton, through which energy is transferred upward.

### 3.3. Change in Energy Conversion Efficiency

**Figure 5:**
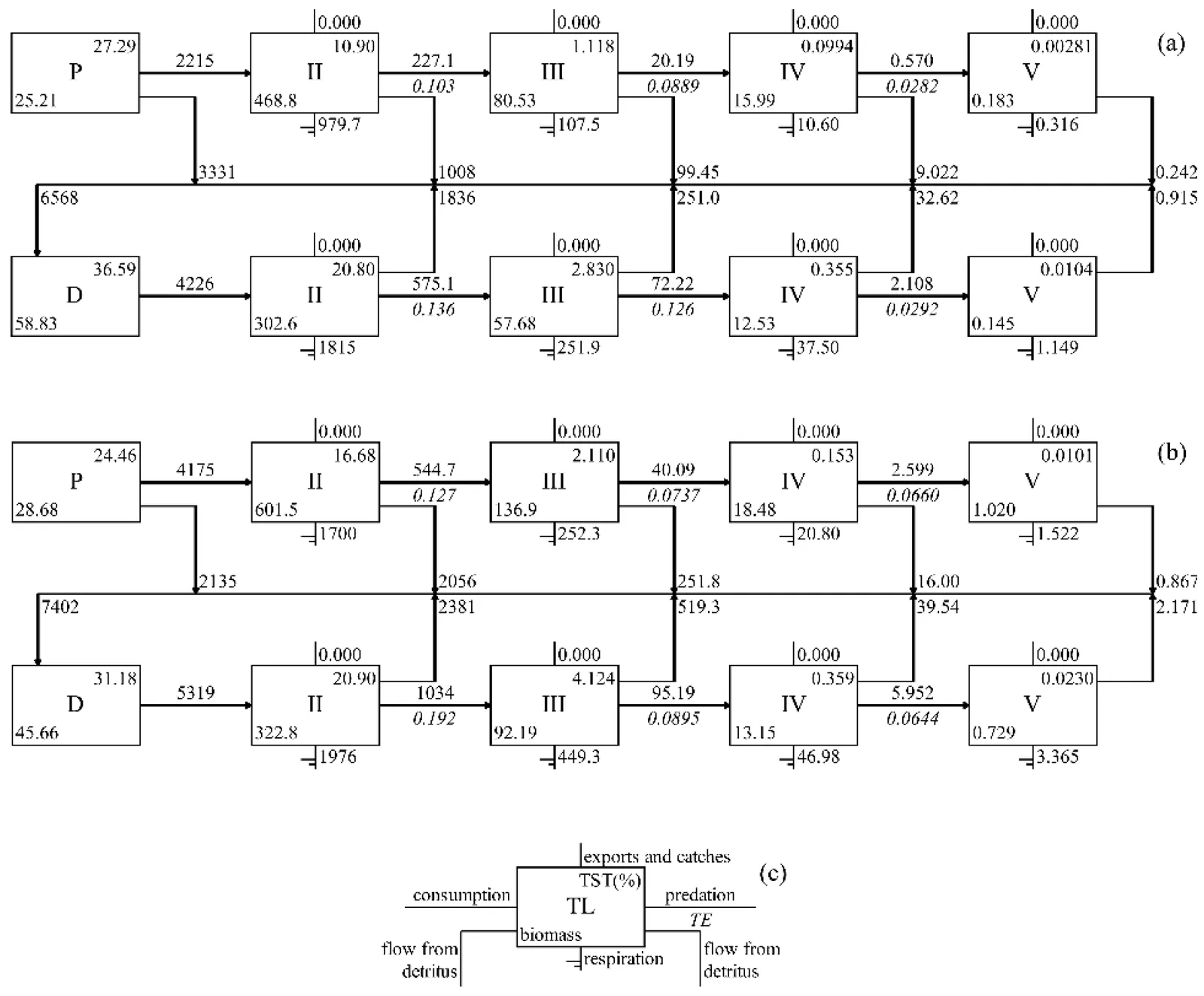
(**a**) The Lindeman energy flow network diagram for the reef deployment area in 2019. (**b**) The Lindeman energy flow network diagram for the reef deployment area in 2022. (**c**) The schematic diagram of a single trophic level in the Lindeman energy-flow network. Notes: The direction of the arrows indicates the flow direction of energy. The numbers above the arrows are the quantity of energy transferred, and those below are the energy transfer efficiency; P is primary producers; D is detritus; II–V are trophic levels 2–5. The numbers in the upper right corner of the squares are the proportion of the energy of the corresponding trophic level in the total system flow. The numbers in the lower left corner represent the biomass of the trophic level. The numbers above the squares are the output and catch, and those below are the energy for respiration.

In 2019 and 2022, the energy flow primarily involved five aggregate trophic levels, which are shown in Figure 5. The energy flow within the system in both years was primarily concentrated between trophic levels I and II, followed by between trophic levels II and III. As the trophic level increased, the energy flow gradually decreased. In 2022, the proportion of energy flow at trophic level II in the total flow increased from 31.70% to 37.58%, while the energy flow in trophic level III increased from 3.948% to 6.234% of the total system flow. The energy flow proportion in trophic level IV rose from 0.4544% to 0.512%, and that in trophic level V increased from 0.0132% to 0.0331%.

The energy transfer efficiencies at each trophic level in the reef deployment area in 2019 and 2022 are shown in Table 4 and Table 5. In 2022, the proportion of the total flow originating from detritus in the reef deployment area decreased from 61.0% to 57%. The average transfer efficiency of primary producers increased from 6.631% to 8.507%. The average transfer efficiency of detritus increased from 7.930% to 10.34%. The overall total transfer efficiency of the system increased from 7.464% to 9.613%. The transfer efficiencies of the grazing food chain and the detrital food chain were higher in 2022 than in 2019.

### 3.4. Mixotrophic Effects and Keystone Species

**Figure 6:**
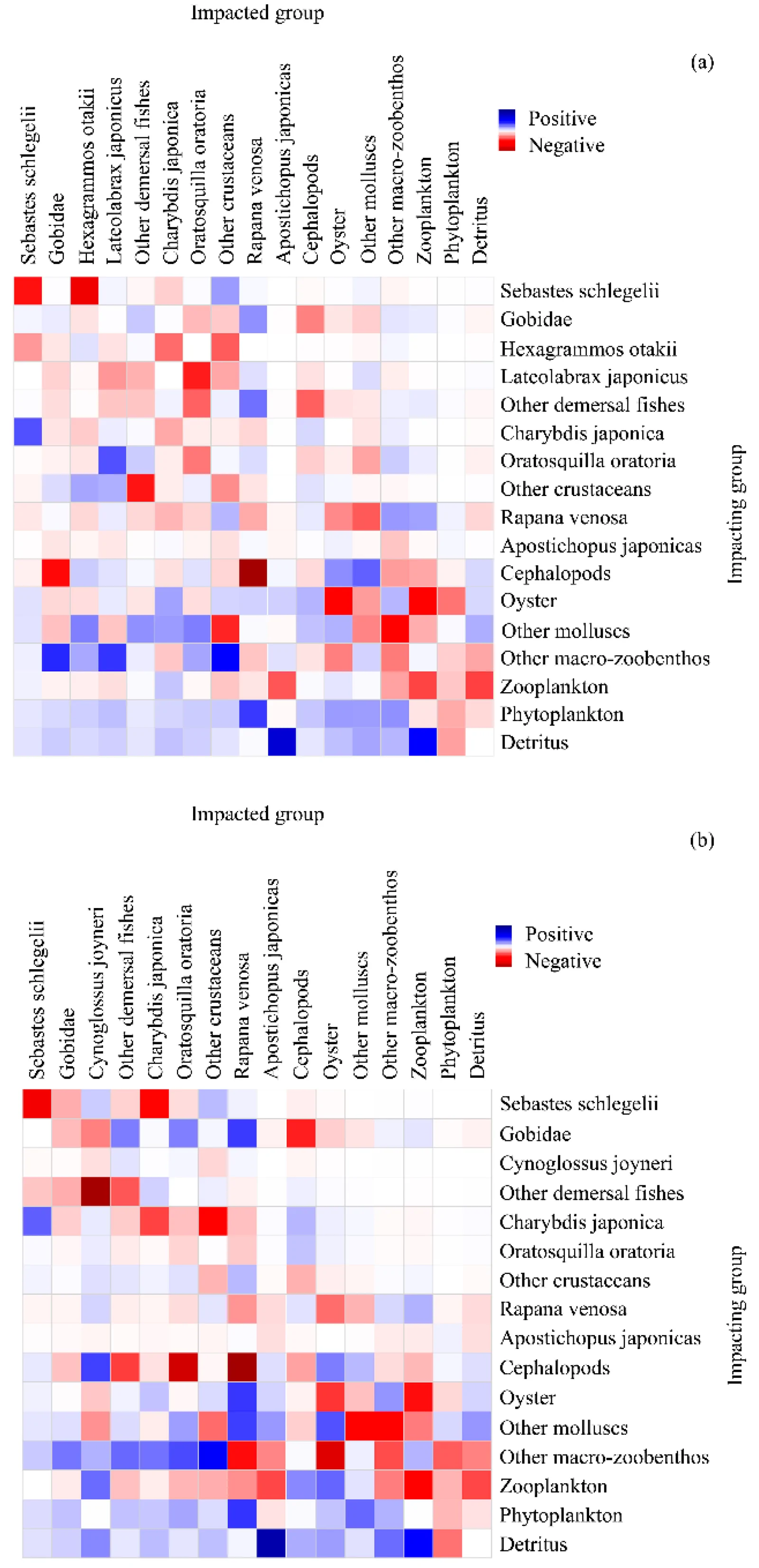
(**a**) The mixed trophic impact in 2019. (**b**) The mixed trophic impact in 2022. Note: The horizontal axis represents impacted functional groups, and the vertical axis represents impacting functional groups. Blue indicates a positive impact, while red indicates a negative impact. The intensity of the color reflects the strength of the impact.

The results of the mixotrophic effects in the reef deployment area in 2019 and 2022 are shown in Figure 6. Generally, an increase in prey abundance has a positive impact on predators, while conversely, an increase in predator abundance has a negative effect on prey. In the 2019 reef deployment area, the predator *Sebastes schlegelii* had a significant negative effect on *Charybdis japonica*. Gobiidae exerted very pronounced negative effects on cephalopods, other demersal fishes, *Cynoglossus joyneri*, and *Charybdis japonica* on other crustaceans. In 2022, *Sebastes schlegelii* had negative impacts on *Hexagrammos otakii*, *Lateolabrax japonicus* on *Oratosquilla oratoria*, and cephalopods on Gobiidae and *Rapana venosa*. Similarly, large benthic organisms, serving as prey species in the two-year reef restoration area, exert varying degrees of positive influence on higher trophic level fish and crustaceans. Phytoplankton and detritus exert positive effects on most functional groups.

Except for detritus, other functional groups had varying degrees of negative impacts on themselves, such as *Sebastes schlegelii*, other demersal fish, and other mollusca, which indicates the existence of intraspecific competition. Additionally, functional groups with similar diets exerted negative effects on each other due to interspecific competition.

**Figure 7:**
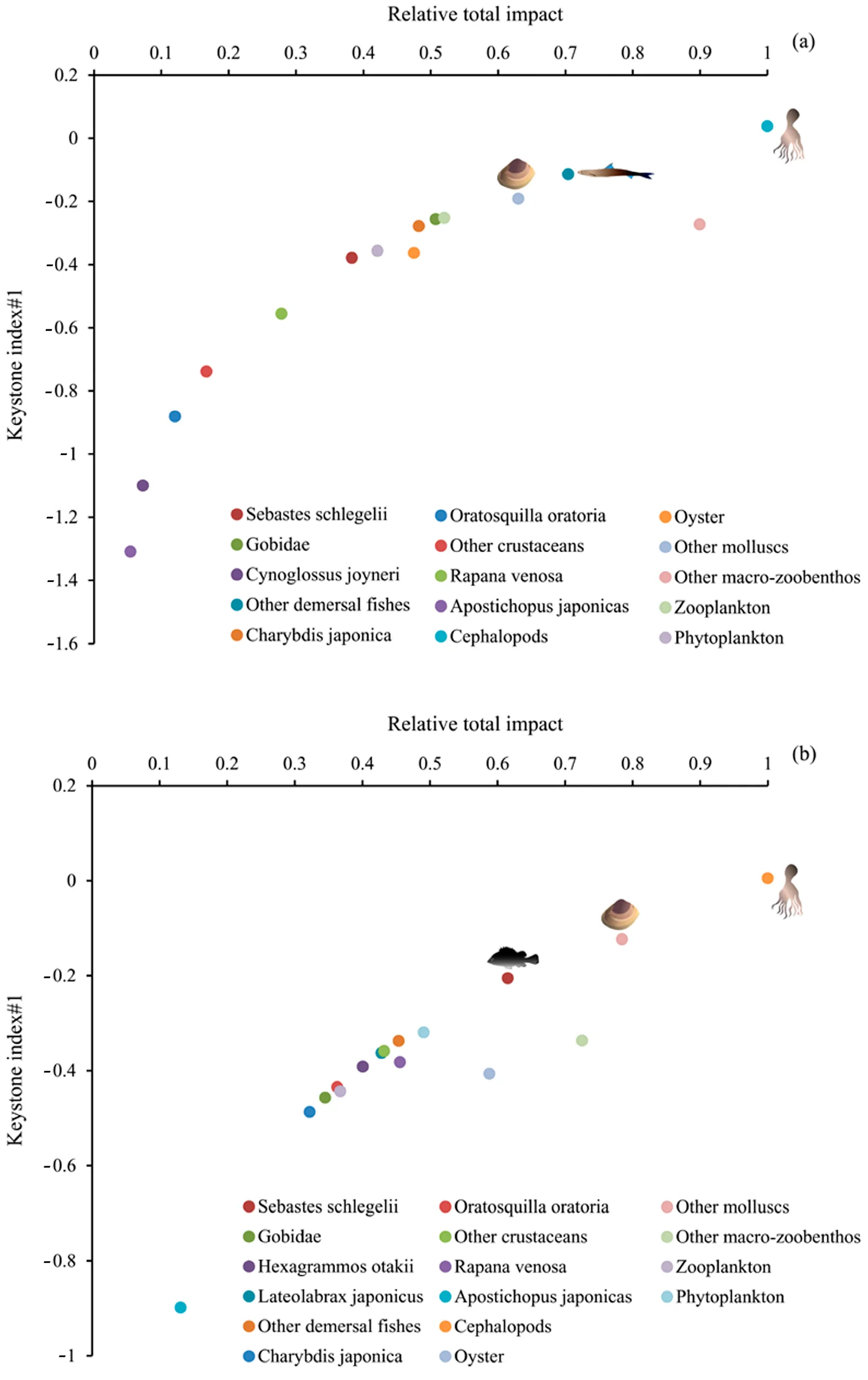
(**a**) The distribution of the keystone index in 2019. (**b**) The distribution of the keystone index in 2022. Note: The horizontal axis represents relative total impact, and the vertical axis represents the keystone index. We selected keystone index 1 as the subject of investigation. Keystone index #1 refers to the first keystone index provided in the model.

Keystone indexes and relative total impact were obtained through keystone species analysis, as shown in Figure 7. In 2019, the top three keystone species were cephalopods, other demersal fish, and other mollusca. In 2022, they were cephalopods, other mollusca, and *Sebastes schlegelii*. During the continuous conservation process, cephalopods ranked first in terms of keystone index and relative total impact, indicating that they stably play an important role in the biological community structure of this ecosystem.

## 4. Discussion

### 4.1. Trophic Structure Characteristics

The nutritional structure is a direct manifestation of the function of an ecosystem [51]. Trophic level I includes detritus and phytoplankton and is the main source of nutrients and energy for the entire ecosystem. Phytoplankton account for 50% of global primary productivity and are the nutritional lifeline of the aquatic food chain [52]. In 2022, phytoplankton biomass was 13.78% higher than in 2019. The reason for the increase might be that in the marine ranch environment, the deployment of artificial reefs would change the hydrodynamic conditions, promote the mixing and distribution of nutrients, and be conducive to the growth of phytoplankton [53]. This may have further increased the biomass of primary producers in the ecosystem. This study suggests that sustained resource conservation may be one factor contributing to increased total primary production in the Furong Island marine ranching ecosystem, thereby providing a broader energy and nutrient basis for a more complex food-web structure.

Data showed that from 1950 to 1994, the average trophic level of global catches decreased from 3.3 to 2.1, and the catches shifted from bottom-dwelling fish with high trophic levels and long lifespans to middle-upper layer fish with low trophic levels and short lifespans [54]. Excessive fishing pressure can change the genetic structure of fish populations, resulting in smaller individuals with early sexual maturity being more adapted to anthropogenic selection pressure and dominating the population [55]. Studies have shown that the deployment of artificial reefs has improved the ecological environment, providing good shelter and abundant food sources for fish. At the same time, it has also brought a better conservation environment for organisms in the reef area, increasing the trophic levels of organisms such as *Sebastes schlegelii* and *Hexagrammos otakii* [56]. During the sustained conservation of the marine ranching ecosystem, trophic level II included mollusca, benthic organisms, and zooplankton, which played key mediating roles in material transfer and energy flow [57]. Trophic level III includes crustaceans, cephalopods, and some fish. At trophic level IV, only *Sebastes schlegelii* and *Lateolabrax japonicus* were found, which is consistent with the research results of Yuan et al. on the artificial reef area of Laizhou Bay [3].

In this study, the mean trophic level of the Furong Island marine ranching ecosystem increased from 2.050 to 2.159, and most functional groups showed slightly higher trophic levels in 2022 than in 2019, with several groups exhibiting markedly higher estimated biomass. Although the same sampling methods were applied in both survey periods, the observed biomass differences may have been influenced to some extent by sampling efficiency and natural interannual variability. Furthermore, the functional group composition differed between the 2019 and 2022 models because the species composition recorded during the two surveys was not identical, and this difference may also have contributed to the variation in some trophic and network indices. Additional uncertainty arises from the fact that some P/B, Q/B, and diet parameters were derived from previous studies, and the two models were balanced independently.

Environmental changes and human intervention can alter the feeding strategies of organisms within an ecosystem, resulting in shifts in trophic levels, and potentially leading to either degradation or upgrading [13]. Research indicated that the enhanced release of filter-feeding bivalves in the nearshore waters of Tangshan had increased bivalve biomass, which in turn has changed the predation strategies of certain predators and altered the trophic status of some predators [58]. Canadian lakes were invaded by smallmouth bass (*Micropterus dolomieu*) and rock bass (*Ambloplites rupestris*). This led to a shift in the predation strategy of the local top predator, lake trout, from a fish-dominated diet to one dominated by plankton. The fish consumption proportion decreased significantly from an average of 62% to 22%, resulting in a notable reduction in trophic levels [59]. In this study, the species in the marine ranch changed between 2019 and 2022. For instance, *Sebastes schlegelii* and *Cynoglossus joyneri* preyed on other demersal fishes in 2019, but *Cynoglossus joyneri* did not appear in the survey data in 2022. Moreover, due to the change in the composition of other demersal fishes, these two species, *Cynoglossus joyneri* and *Sebastes schlegelii*, were no longer included in the diet composition of other demersal fishes. However, due to the change in the proportion of various feeds, predation pressure on higher-trophic-level organisms increased; thus, the trophic levels did not decrease but increased instead.

### 4.2. Changes in Energy Flow Through Ecosystems

During the three-year interval between the two surveys, total energy flow within the Furong Island marine ranching ecosystem occurred predominantly across the first five trophic levels. The energy transfer efficiency was highest between trophic levels II and III, reaching 12.46% in 2019 and 16.28% in 2022. Between trophic levels IV and V, transfer efficiency increased from 2.897% in 2019 to 6.484% in 2022, indicating more efficient energy transfer to higher trophic levels in the 2022 model. The total transfer efficiency also increased from 7.464% in 2019 to 9.613% in 2022. Although the 2022 value remained slightly below 10%, it was close to the commonly cited reference value of approximately 10% for trophic energy transfer in aquatic ecosystems [60]. Furthermore, the value of 9.613% was slightly higher than the overall average transfer efficiency of 9.2% reported by Christensen and Pauly for 41 aquatic ecosystems [61]. These changes generally suggest a tendency toward greater ecosystem maturity in 2022.

A quantitative analysis of energy flow in the reef area of Furong Island marine ranch revealed a dominance of detritus food chain characteristics, consistent with many coastal ecosystems [62]. The upwelling from rocky reefs enhances consumers’ contact with sediment detritus, making the impact of the detrital food chain on the rocky reef ecosystem more obvious compared to nearshore bare substrate systems [63]. Detritus energy flow accounted for 61.0% and 57.0% of the ecosystem, respectively. Detrital energy accounted for a relatively high proportion of the total energy flow of the Furong Island marine ranch ecosystem, suggesting a greater reliance on detritus energy during energy circulation. Supplemented by terrestrial inputs and aquaculture detritus, nearshore marine ranches have established substantial detritus reservoirs, which have fostered a proliferation of detritivores (such as Bivalvia and Polychaeta). As their biomass increases, these organisms provide a more extensive energy base for higher trophic level organisms [62,64]. It is important to note that this model does not account for the utilization of detritus by microorganisms, potentially resulting in an underestimation of the actual transfer efficiency [62].

### 4.3. Ecosystem Maturation Trends

The ratio of total primary productivity to total respiration (TPP/TR) serves as a crucial indicator of ecosystem maturity [49]. During the initial stages of ecological succession, TPP/TR > 1 signifies that the system is accumulating biomass through net photosynthesis. As the ecosystem matures, the TPP/TR value gradually approaches 1, indicating a dynamic equilibrium between production and consumption [13,65]. At this stage, TPP/TR remains relatively low, while TB/TST increases to a comparatively high level, reflecting enhanced energy utilization efficiency and structural stability within the system. In this study, the TPP/TR value of the Furong Island marine ranch decreased from 1.731 in 2019 to 1.418 in 2022, indicating a tendency toward greater ecosystem maturity in the 2022 model. Ju et al. compared the ecosystems of Laizhou Bay and Haizhou Bay based on their food webs [45], revealing that the TPP/TR value for Laizhou Bay during 2014–2015 was 2.995. In a study conducted by Ding et al. on the ecological capacity of *Apostichopus japonicus* in the marine ranch of Furong Island, the TPP/TR value for Furong Island in 2019 was determined to be 1.454. The TPP/TB decreased from 9.388 in 2019 to 8.020 in 2022, and the Shannon diversity index showed only a slight increase, rising from 1.544 to 1.570, which is also consistent with a tendency toward greater ecosystem maturity [13]. Although CI decreased slightly from 0.356 in 2019 to 0.352 in 2022, the number of actual trophic links increased from 80 to 90. This slight decline in CI can be explained, at least in part, by the increase in model resolution, as the number of potential trophic links increased from 225 to 256 with the addition of functional groups. Therefore, the small decrease in CI should not be interpreted as direct evidence of reduced trophic interactions or food-web complexity. Collectively, the observed changes in TPP/TR, TPP/TB, FCI, MPL, SOI, CI, and energy transfer efficiency are consistent with a general trend towards increased ecosystem maturity and more efficient internal energy utilization in the Furong Island marine ranching ecosystem. Nevertheless, the responses were not uniform across all indicators, suggesting that ecosystem structure and functioning remain subject to ongoing adjustment and successional dynamics.

## 5. Conclusions

This study compared the Ecopath models of the Furong Island marine ranching ecosystem in 2019 and 2022 to assess temporal changes in trophic structure, ecosystem attributes, energy transfer, and species interactions. Compared with 2019, the 2022 model showed higher biomass for most functional groups, slightly higher trophic levels, greater total system throughput, and increased energy transfer efficiency. Changes in TPP/TR, TPP/TB, FCI, MPL, SOI, CI, and transfer efficiency generally indicated that, although not all ecosystem indicators exhibited consistent patterns, the ecosystem showed an overall tendency toward greater maturity and more efficient internal energy utilization. The detrital food chain remained the dominant energy pathway, while the contribution of primary producers to ecosystem energy flow increased. Changes in mixed trophic impacts and keystone species further indicated temporal shifts in species interactions between 2019 and 2022. Overall, the changes observed between 2019 and 2022 were consistent with potential ecological improvements under sustained conservation and management. However, because this study was based on only two survey periods without a reference area, and the two Ecopath models differed to some extent in functional group composition, factors such as interannual environmental variability, recruitment dynamics, and other management activities may also have contributed to the observed changes. Therefore, further studies are needed to disentangle the potential contributions of these factors and to provide a more detailed assessment of the observed temporal differences.

## Figures and Tables

**Table 1:** Functional groups and main species composition of the ecosystem model in the reef deployment area of Furong Island marine ranching in 2019 and 2022.

Group Code	Group Name	2019	2022
1	*Sebastes schlegelii*	*Sebastes schlegelii*	*Sebastes schlegelii*
2	Gobiidae	*Chaeturichthys stigmatias*, *Acanthogobius ommaturus*	*Chaeturichthys stigmatias*, *Acanthogobius ommaturus*, *Tridentiger trigonocephalus*
3	*Hexagrammos otakii*	-	*Hexagrammos otakii*
4	*Cynoglossus joyneri*	*Cynoglossus joyneri*	-
5	*Lateolabrax japonicus*	-	*Lateolabrax japonicus*
6	Other demersal fishes	*Callionymus beniteguri*, *Saurida elongate*, *Sillago sihama*	*Conger myriaster*, *Kareius bicoloratus*, *Johnius belengeri*, *Platycephalus indicus*
7	*Charybdis japonica*	*Charybdis japonica*	*Charybdis japonica*
8	*Oratosquilla oratoria*	*Oratosquilla oratoria*	*Oratosquilla oratoria*
9	Other crustacean	*Lysmata vittata*, *Trachypenaeus curvirostris*, *Crangon hakodatei*, *Portunus trituberculatus*, *Heikeopsis japonica*	*Lysmata vittata*, *Exopalaemon carinicauda*, *Palaemon graviera*, *Trachysalambria curvirostris*, *Portunus trituberculatus*, *Pugettia quadridens*
10	*Rapana venosa*	*Rapana venosa*	*Rapana venosa*
11	*Apostichopus japonicus*	*Apostichopus japonicus*	*Apostichopus japonicus*
12	Cephalopods	*Octopus* *Ocellatus*, *Loligo chinensis*	*Octopus variabilis*, *Loligo chinensis*, *Octopus ocellatus*
13	Oyster	Oyster	Oyster
14	Other Mollusca	*Glossaulax didyma*, *Theora fragilis*, *Ringicula doliaris*, *Potamocorbula laevis*, *Moerella jedoensis*, *Nucula paulula*	*Glossaulax didyma*, *Theora fragilis*, *Ringicula doliaris*, *Potamocorbula laevis*, *Nitidotellina minuta*, *Reticunassa festiva*
15	Other macro-zoobenthos	*Eteone longa*, *Glycera chirori*, *Scoletoma longifolia*, *Potamilla* sp., *Tharyx* sp., *Aricidea fragilis*, *Sigambra* sp., *Glycinde gurjanovae*, *Nephthys oligobranchia*, *Chaetozone setosa*, *Scolelepis squamata*, *Harmothoinae*, *Paralacydonia paradoxa*, *Schistomeringos japonica*, *Anaitides papillosa*, *Ampharete acutifrons*, *Aricidea fragilis*, *Cirriformia tentaculate*, *Ophiodromus anguotifrons*, *Lumbrinereis heteropoda*, *Mediomastus* sp., *Spiochaetopterus* sp., *Prionospio* sp., *Philyra pisuma*, *Ampelisca brevicornis, Hemileucon bidentatus*, *Corophium* sp., *Callianasa harmandi*, *Pontocrates altamarimus*, *Caviplaxus jiaozhouwanensis*, *Eocuma lata*, *Ampelisca cyclops*, *Caprella* sp., *Harpiniopsis vadiculus*, *Paranthura japonica*, *Orchomene breviceps*, *Leptochela gracilis*, *Echinocardium cordatum*, *Protankyra bidentata*, *Virgulariidae*, *Nemertinea*	*Eteone longa*, *Armandia intermedia*, *Scoletoma longifolia*, *Potamilla* sp., *Tharyx* sp., *Notomastus latericeus*, *Sigambra* sp., *Glycinde gurjanovae*, *Pseudopolydora*, *Chaetozone setosa*, *Scolelepis squamata*, *Harmothoinae*, *Paralacydonia paradoxa*, *Schistomeringos japonica*, *Anaitides papillosa*, *Ampharete acutifrons*, *Aricidea fragilis*, *Cirriformia tentaculate*, *Ophiodromus anguotifrons*, *Lumbrinereis heteropoda*, *Mediomastus* sp., *Glycera subaenea*, *Prionospio* sp., *Philyra pisuma*, *Ampelisca brevicornis*, *Hemileucon bidentatus*, *Corophium* sp., *Callianasa harmandi*, *Pontocrates altamarimus*, *Caviplaxus jiaozhouwanensis*, *Leptochela gracilis*, *Eocuma lata*, *Ampelisca cyclops*, *Macrophthalmus dilatatum*, *Eucrate crenata*, *Paranthura japonica*, *Orchomene breviceps*, *Echinocardium cordatum*, *Protankyra bidentata*, *Luidia quinaria von Martens*
16	Zooplankton	Others	Others
17	Phytoplankton	Others	Others
18	Detritus	Detritus	Detritus

**Table 2:** Input and output parameters for Ecopath functional groups in the reef deployment area of Furong Island marine ranching in 2019 and 2022.

Functional Group Code	Functional Group	Trophic Level		Biomass (t km^−2^)		P/B (Year)		Q/B (Year)		EE	
		2019	2022	2019	2022	2019	2022	2019	2022	2019	2022
1	*Sebastes schlegelii*	**4.079**	**4.183**	0.0605	0.6537	1.006	1.0056	5.300	5.300	**0.325**	**0.393**
2	Gobiidae	**3.381**	**3.446**	0.0411	0.9410	2.300	2.310	7.880	7.940	**0.579**	**0.764**
3	*Hexagrammos otakii*	**-**	**3.832**	-	0.7032	-	0.820	-	7.280	**-**	**0.701**
4	*Cynoglossus joyneri*	**3.306**	**-**	0.0085	-	1.479	-	4.950	-	**0.359**	**-**
5	*Lateolabrax japonicus*	**-**	**4.058**	-	0.7484	-	0.370	-	4.770	**-**	**0.000**
6	Other demersal fishes	**3.659**	**3.873**	0.0168	0.8157	0.870	0.750	5.380	5.280	**0.500**	**0.686**
7	*Charybdis japonica*	**3.303**	**3.310**	0.0655	1.1667	4.000	4.000	13.50	11.60	**0.803**	**0.931**
8	*Oratosquilla oratoria*	**3.257**	**3.289**	0.0110	0.4940	8.000	8.000	30.00	30.00	**0.244**	**0.601**
9	Other crustacean	**3.245**	**3.272**	0.0107	0.4826	6.100	6.000	20.40	20.00	**0.903**	**0.944**
10	*Rapana venosa*	**2.679**	**2.698**	50.83	80.00	0.260	1.370	2.820	5.310	**0.000**	**0.010**
11	*Apostichopus japonicus*	**2.006**	**2.245**	100.0	40.00	0.600	0.600	3.360	3.370	**0.000**	**0.000**
12	Cephalopods	**3.535**	**3.621**	0.0171	1.1915	2.640	2.180	9.000	7.240	**0.957**	**0.989**
13	Oyster	**2.312**	**2.273**	97.40	269.1	2.892	4.310	10.50	16.63	**0.950**	**0.689**
14	Other Mollusca	**2.411**	**2.417**	38.58	42.0363	6.550	6.550	22.50	22.50	**0.237**	**0.146**
15	Other macrozoobenthos	**2.128**	**2.129**	270.06	294.2537	1.670	1.670	8.350	8.350	**0.198**	**0.493**
16	Zooplankton	**2.000**	**2.000**	8.402	25.43	65.70	38.30	328.5	128.5	**0.959**	**0.986**
17	Phytoplankton	**1.000**	**1.000**	25.206	28.68	220.0	220.0	-	-	**0.399**	**0.662**
18	Detritus	**1.000**	**1.000**	58.83	45.66	-	-	-	-	**0.604**	**0.689**

Note: “-” Indicates no data; bold indicates output parameters.

**Table 3:** Energy flow parameters in the reef deployment area of Furong Island marine ranching in 2019 and 2022.

Parameters	2019	2022	Units
Sum of all consumption (TC)	7387.512	11,775.890	t·km^−2^·a
Sum of all exports (TEX)	2775.192	2402.591	t·km^−2^·a
Sum of all respiratory flows (TR)	3203.967	4450.936	t·km^−2^·a
Total flows into detritus(TDET)	7001.484	7721.711	t·km^−2^·a
Total system throughput (TST)	20,368.160	26,351.131	t·km^−2^·a
Sum of all production (TP)	7156.561	9362.244	t·km^−2^·a
Calculated total net primary production (TPP)	5545.32	6309.600	t·km^−2^·a
Total primary production/total respiration (TPP/TR)	1.731	1.418	t·km^−2^·a
Net system production (NSP)	2341.353	1858.665	t·km^−2^·a
Total primary production/total biomass (TPP/TB)	9.388	8.020	t·km^−2^·a
Total biomass/total throughput (TB/TST)	0.029	0.030	t·km^−2^·a
Total biomass (excluding) (TB)	590.709	786.697	t·km^−2^
Finn’s cycling index(FCI)	15.03	19.11	%
Mean Path Length (MPL)	3.407	3.845	
Connectance Index (CI)	0.356	0.352	
System Omnivory Index(SOI)	0.172	0.189	
Ascendancy(A)	21.95	18.41	%
Total transfer efficiency between TL (TE)	7.464	9.613	%
Shannon diversity index	1.544	1.570	-

**Table 4:** Energy transfer efficiency at different trophic levels in the reef deployment area ecosystem in 2019.

Energy Source	Trophic Level			
	II	III	IV	V
Producer	10.25	8.892	2.822	2.197
Detritus	13.61	12.56	2.919	2.121
All flows	12.46	11.52	2.897	2.137
Proportion of total flow originating from detritus (%)	61.0			
Transfer efficiencies from primary producers (%)	6.361			
Transfer efficiencies from detritus (%)	7.930			
Total transfer efficiencies (%)	7.464			

**Table 5:** Energy transfer efficiency at different trophic levels in the reef deployment area ecosystem in 2022.

Energy Source	Trophic Level			
	II	III	IV	V
Producer	12.66	7.368	6.597	8.083
Detritus	19.17	8.948	6.436	6.675
All flows	16.28	8.413	6.484	7.104
Proportion of total flow originating from detritus (%)	57.0			
Transfer efficiencies from primary producers (%)	8.507			
Transfer efficiencies from detritus (%)	10.34			
Total transfer efficiencies (%)	9.613			

## Data Availability

Data will be made available on request.

## Supplementary Materials

The following supporting information can be downloaded at: https://www.mdpi.com/article/10.3390/biology15181620/s1. Table S1: Diet composition (proportion) of functional groups in Furong Island reef deployment in 2019; Table S2: Diet composition (proportion) of functional groups in Furong Island reef deployment in 2022; Table S3: Diet composition (proportion) of functional groups in Furong Island reef deployment in 2019 (without balanced); Table S4: Diet composition (proportion) of functional groups in Furong Island reef deployment in 2022 (without balanced).
