# Supplementary material for: Evaluation of the Sustainability of Marine Resources in the Reef Areas of Furong Island Marine Ranching Based on the Ecopath Model
Source: Biology (Basel). 2026 Sep 15;15(18):1620. doi: 10.3390/biology15181620 (PMC13604485; doi:10.3390/biology15181620)
Supplement: Supplementary file 1 [file biology-15-01620-s001.zip › biology-4481884-supplementary.pdf]

Table S1 Diet composition (proportion) of functional groups in Furong Island reef deployment in 2019

Prey \ predator	1	2	3	4	5	6	7	8	9	10	11	12	13	14
Sebastes schlegelii	0.0464			0.0544										
Gobiidae	0.0575	0.027	0.003	0.135		0.001				0.0972				
Cynoglossus joyneris				0.05										
Other demersal fishes	0.0053			0.0234						0.0227				
Charybdis japonica	0.553				0.0247					0.0718				
Oratosquilla oratoria	0.0103	0.0035		0.0007			0.0039			0.105				
Other crustacean	0.036		0.1	0.001	0.0384		0.0401			0.0026				
Rapana venosa										0.0264				
Cephalopods	0.01	0.07	0.03	0.0447			0.0305			0.0349				
Oyster					0.121			0.405		0.0744	0.005		0.0906	
Other Mollusca	0.226	0.44	0.21	0.341	0.308	0.501	0.105	0.105		0.0314		0.0508		
Other macrozoobenthos	0.055	0.4	0.352	0.35	0.459	0.39	0.803		0.005	0.212		0.1		
Zooplankton		0.0595	0.305		0.0494	0.109	0.0139			0.322	0.305	0.226	0.0092	
Phytoplankton							0.0012	0.285			0.295	0.502	0.392	0.2
Detritus							0.0019	0.205	0.995		0.395	0.121	0.508	0.8
Import	0	0	0	0	0	0	0	0	0	0	0	0	0	0
Sum	1	1	1	1	1	1	1	1	1	1	1	1	1	1

Note: *Apostichopus japonicus* were not included in the diet composition table S1 because it had no predators in this model.

Table S2 Diet composition (proportion) of functional groups in Furong Island reef deployment in 2022

Prey \ predator	1	2	3	4	5	6	7	8	9	10	11	12	13	14	15
Sebastes schlegelii	0.0552		0.0131												
Gobiidae	0.0268	0.007	0.015	0.0676	0.0777		0.0017				0.0972				
Hexagrammos otakii	0.117														
Other demersal fishes	0.001		0.009	0.0188	0.005			0.0202			0.01				
Charybdis japonica	0.55		0.253	0.0394	0.0017	0.01					0.1				
Oratosquilla oratoria	0.01	0.02	0.0132	0.352	0.188			0.0019			0.0047				
Other crustacean	0.008	0.0505	0.18	0.154	0.0212	0.02		0.0491			0.0026				
Rapana venosa											0.126				
Cephalopods	0.001	0.1	0.001	0.119	0.222			0.0118			0.0369				
Oyster						0.495			0.503		0.194	0.0742		0.0996	
Other Mollusca	0.113	0.3	0.416		0.201	0.315	0.56	0.112	0.0406		0.231		0.0018		
Other macrozoobenthos	0.118	0.454	0.1	0.25	0.283	0.04	0.41	0.804		0.217	0.184		0.202		
Zooplankton		0.0685				0.12	0.0287	0.0007			0.0127	0.174	0.186	0.0022	
Phytoplankton									0.383			0.454	0.39	0.39	0.2
Detritus									0.0729	0.783		0.248	0.22	0.508	0.8
Import	0	0	0	0	0	0	0	0	0	0	0	0.05	0	0	0
Sum	1	1	1	1	1	1	1	1	1	1	1	1	1	1	1

Note: *Lateolabrax japonicus* and *Apostichopus japonicus* were not included in the diet composition table S2 because they had no predators in this model.

Table S3 Diet composition (proportion) of functional groups in Furong Island reef deployment in 2019 (without balanced)

Prey \ predator	1	2	3	4	5	6	7	8	9	10	11	12	13	14
Sebastes schlegelii	0.0464			0.154										
Gobiidae	0.0675			0.545						0.0972				
Cynoglossus joyneris														
Other demersal fishes	0.0103			0.113						0.0227				
Charybdis japonica	0.603				0.0247					0.272				
Oratosquilla oratoria	0.0103						0.0039			0.0047				
Other crustacean	0.036				0.0494		0.0401			0.0026				
Rapana venosa										0.126				
Cephalopods				0.0447			0.0305			0.0369				
Oyster								0.503		0.194	0.0742		0.0196	
Other Mollusca	0.226	0.394	0.0231	0.0212	0.778	0.562	0.105	0.0566		0.231				
Other macrozoobenthos		0.527	0.962	0.122	0.0988	0.39	0.803		0.217	0.0199				
Zooplankton		0.0795	0.0151		0.0494	0.0487	0.0139				0.144	0.186		
Phytoplankton							0.0012	0.377			0.514	0.592	0.392	0.4
Detritus							0.0019	0.0629	0.783		0.268	0.222	0.588	0.8
Import	0	0	0	0	0	0	0	0	0	0	0	0	0	0
Sum	1	1	1	1	1	1	1	1	1	1	1	1	1	1

Note: *Apostichopus japonicus* were not included in the diet composition table S1 because it had no predators in this model.

Table S4 Diet composition (proportion) of functional groups in Furong Island reef deployment in 2022(without balanced)

Prey \ predator	1	2	3	4	5	6	7	8	9	10	11	12	13	14	15
Sebastes schlegelii	0.0452		0.0131												
Gobiidae	0.0268		0.015	0.0376	0.0677						0.0972				
Hexagrammos otakii	0.267														
Other demersal fishes			0.03	0.0188	0.0521			0.0202			0.0227				
Charybdis japonica	0.733		0.153	0.0094	0.0017	0.01					0.272				
Oratosquilla oratoria	0.01			0.662	0.288			0.0019			0.0047				
Other crustacean	0.01		0.193	0.154	0.121	0.02		0.0691			0.0026				
Rapana venosa											0.126				
Cephalopods				0.119	0.332			0.0118			0.0369				
Oyster						0.595			0.503		0.194	0.114		0.0196	
Other Mollusca	0.113	0.105	0.516		0.0013	0.315	0.562	0.0119	0.0566		0.231				
Other macrozoobenthos	0.0350	0.827	0.08		0.136	0.04	0.390	0.884		0.217	0.0119				
Zooplankton		0.0685				0.02	0.0487	0.0007				0.144	0.186		0.2
Phytoplankton									0.377			0.514	0.592	0.392	0.8
Detritus									0.0629	0.783		0.228	0.222	0.588	
Import	0	0	0	0	0	0	0	0	0	0	0	0.00	0	0	0
Sum	1	1	1	1	1	1	1	1	1	1	1	1	1	1	1

Note: *Lateolabrax japonicus* and *Apostichopus japonicus* were not included in the diet composition table S2 because they had no predators in this model.
